# Mechanobiological Analysis of Nanoparticle Toxicity

**DOI:** 10.3390/nano13101682

**Published:** 2023-05-19

**Authors:** Abdurazak Aman Ketebo, Shahab Ud Din, Gwang Lee, Sungsu Park

**Affiliations:** 1 School of Mechanical Engineering, Sungkyunkwan University, Suwon 16499, Republic of Korea; 2 Department of Biophysics, Institute of Quantum Biophysics, Sungkyunkwan University, Suwon 16419, Republic of Korea; 3 Department of Physiology, Ajou University School of Medicine, Suwon 16499, Republic of Korea; 4 Department of Molecular Science and Technology, Ajou University, Suwon 16499, Republic of Korea

**Keywords:** mechanobiology, nanoparticle, nanotoxicity, rigidity sensing, traction force

## Abstract

Nanoparticles (NPs) are commonly used in healthcare and nanotherapy, but their toxicity at high concentrations is well-known. Recent research has shown that NPs can also cause toxicity at low concentrations, disrupting various cellular functions and leading to altered mechanobiological behavior. While researchers have used different methods to investigate the effects of NPs on cells, including gene expression and cell adhesion assays, the use of mechanobiological tools in this context has been underutilized. This review emphasizes the importance of further exploring the mechanobiological effects of NPs, which could reveal valuable insights into the mechanisms behind NP toxicity. To investigate these effects, different methods, including the use of polydimethylsiloxane (PDMS) pillars to study cell motility, traction force production, and rigidity sensing contractions, have been employed. Understanding how NPs affect cell cytoskeletal functions through mechanobiology could have significant implications, such as developing innovative drug delivery systems and tissue engineering techniques, and could improve the safety of NPs for biomedical applications. In summary, this review highlights the significance of incorporating mechanobiology into the study of NP toxicity and demonstrates the potential of this interdisciplinary field to advance our knowledge and practical use of NPs.

## 1. Introduction

Nanoparticles (NPs) are materials that are less than 100 nm in size [1,2] and are widely used in various biomedical applications, such as biosensors [3,4], transfection agents [5], and cancer treatment [6,7], due to their modifiable material properties. Magnetic NPs (MNPs) coated with biocompatible materials are used in biomedical applications as contrast agents in magnetic resonance imaging (MRI)-based cell labeling, cell tracking, cell separation, and drug delivery vehicles [8,9,10,11,12]. For example, silica-coated magnetic nanoparticles MNPs@SiO_2_(RITC) are synthesized MNPs consisting of a cobalt ferrite core, CoFe_2_O_3_, and a silica shell surrounded by rhodamine B isothiocyanate (RITC) and are used for cell labeling and tracking in vivo [13].

Despite their benefits in biomedical applications, NPs pose potential risks to human health when accidentally entering the human body. These risks include [14] respiratory and cardiovascular problems [15] and neurodegenerative diseases [16,17] due to their nanoscale physicochemical properties, such as a large surface-to-volume ratio, Fenton reaction, and integration of organic compounds [18,19,20]. One of the main reasons that contribute to the toxicity of NPs is their reactive surface area [21], which induces free radical reactive oxygen species (ROS) [22,23] that cause inflammation, endoplasmic reticulum stress, decreased proteasome activity, and disruption of cellular metabolism [24,25]. In particular, NPs induce ROS production, which in turn leads to lipid peroxidation and abnormalities in the cellular mechanisms and cytoskeleton [26].

NPs also cause toxicity in the cellular cytoskeleton by altering its organization, decreasing cell membrane fluidity, and damaging cytoskeletal proteins [27]. When NPs enter the cell via endocytosis, they disrupt the actin structure and damage cellular tight junctions of the cells [28]. After internalization of NPs into the cell, ROS production increases by more than 50%, inducing lipid peroxidation and resulting in decreased cell membrane fluidity [28]. Furthermore, NPs alter cytoskeletal components such as fascin (FSCN1) and paxillin (PXN) [28]. These toxic effects of NPs on the cell cytoskeleton can change biochemical signals that affect the cell’s mechanobiological responses, such as traction force (TF) production and rigidity sensing. These responses determine essential cellular functions such as cell migration, division, and proliferation [29,30].

**Figure 1:**
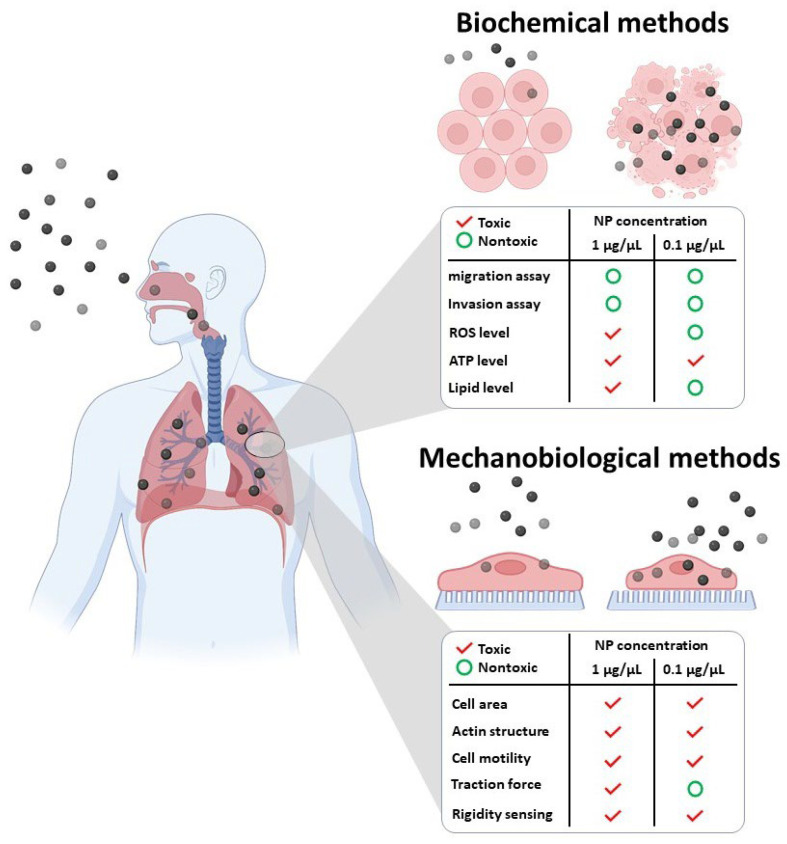
Schematic diagram illustrating the various biochemical and mechanobiological methods used to study nanotoxicity.

Various methods have been developed to assess the toxicity of NPs, as shown in Figure 1. Biochemical methods such as ATP, ROS, and lipid production levels have been used to demonstrate the toxicity of MNPs@SiO_2_(RITC) in multiple cells at higher concentrations of 1 µg/µL. Migration and invasion assays have also been used to investigate NP toxicity [25,31]. However, these methods may not be sensitive enough to detect the toxic effects of MNPs@SiO_2_(RITC) even at concentrations of 1 µg/µL. On the contrary, mechanobiological analyses, such as cell surface area, cell aspect ratio, length of filopodia, focal adhesion area, TF production, and stiffness detection measurements, are more sensitive to detecting the toxicity of NPs below 1 µg/µL [31,32]. Biochemical assays that are used to study the effects of NPs have typically been applied to collective cells and may not be sensitive enough to detect NP concentrations below 1 µg/µL. In contrast, mechanobiological methods that are used to study the behavior of individual cells are more sensitive in detecting NP toxicity but require advanced equipment and skilled personnel. As a result, there have been relatively few studies on nanotoxicity that have utilized mechanobiology methods.

A variety of technology platforms and assessment systems have been developed to analyze the mechanical and physical changes that occur at the cellular level, allowing for the analysis of biophysical phenotypes. These changes can provide insights into how mechanical signals affect the biological and functional responses of cells to NP treatment [31,33,34]. Methods used to study cell biophysical responses include atomic force microscopy, micropipette aspiration, uniaxial stretcher, and optical and magnetic tweezers [31,35,36]. Advanced techniques, such as tensile force microscopy, have been developed to analyze the mechanical forces between cells and their surrounding matrix using soft elastic gel substrates [37,38]. Recently, submicron elastomeric pillars made of polydimethylsiloxane (PDMS) have been developed to detect nanometer-levels of cellular traction forces [31,39,40] and rigidity sensing [32,41,42]. These techniques incorporate microscopy to study cells treated with NPs [31,42].

The mechanobiological response elicited by biomechanical signals from the external environment is essential for regulating normal cell functions. Altered mechanobiological responses can lead to diseases such as cancer, asthma, and heart disease [43], highlighting the importance of studying the mechanobiological effects of NPs on cells. To investigate these effects, various methods have been employed, including the use of PDMS pillars to study cell motility, TF production, and rigidity sensing contractions of a cell. Soft and rigid PDMS surfaces have also been utilized in conjunction with cell morphological analysis to study a cell’s rigidity sensing ability. Mechanobiological methods focus on early events of cell attachment, spreading, and motility, making them a fast and sensitive approach for studying the toxicity of NPs.

This review provides an overview of the toxicity of various NPs on the components of the cell cytoskeleton, including their corresponding toxic concentrations. Additionally, we examine the effects of MNPs@SiO_2_(RITC) on cell mechanobiology, specifically in terms of cell motility, TF production, and rigidity sensing. Given the limited research on the toxic effects of NPs in the field of mechanobiology, this review focuses exclusively on MNPs@SiO_2_(RITC) and its potential implications. By exploring the toxic effects of NPs on cell mechanobiology, this review aims to shed light on the broader impacts of NPs on cell function and to provide insights into new ways of analyzing NP toxicity to make them safer for biomedical applications.

## 2. Interaction of NPs with the Cell Cytoskeleton

The absorption parameters of NPs are influenced by particle size, sedimentation, agglomeration, and diffusion [44]. Once inside the cell, NPs can interact with the cell cytoskeleton both directly during internalization and indirectly by inducing ROS that affect cytoskeletal components via a reduction in ATP, alterations in lipid levels, and changes in the expression of cytoskeletal proteins [45]. The initial contact between NPs and the cellular cytoskeleton occurs when NPs are internalized into the cell through the clathrin- and caveolae-mediated endocytosis pathways [46]. These pathways involve various components of the cytoskeleton, such as actin, plasma membrane receptors, clathrin, and adapter proteins [47]. After internalization, NPs can induce oxidative stress, ER stress, altered cellular metabolism, altered oxidative proteins, changes in redox regulation, and mitochondrial dysfunction [24,26,48,49]. Specifically, oxidative stress can inhibit various components of the cytoskeleton, such as lamellipodia and filopodia [6].

### 2.1. Direct Toxic Effect of NPs on Cell Cytoskeleton

Table 1 provides a summary of extensive research investigating the direct toxic effects of various NPs on the cell cytoskeleton. For example, exposure to metal oxide NPs such as TiO_2_ can cause changes in the cytoskeleton, leading to the breakdown of the actin and tubulin network [27,50]. Proteomic research after TiO_2_ exposure has shown certain changes in proteins associated with cytoskeletal disruptions, particularly those involved in cell motility. Similarly, carbon black NPs can lead to dysregulation of migratory cell proteins under similar experimental conditions [50]. However, the patterns of proteome alterations are dissimilar, suggesting that each type of NP causes changes in a biological pathway through different components of the cell migratory protein network [50]. Furthermore, microarray investigations of the human lung epithelial cell line BEAS-2B treated with TiO_2_ NPs have revealed changes in cytoskeleton-related mRNA and miRNA expression, as well as changes in cell adhesion [27]. These findings indicate that NPs can induce changes in gene expression and adhesion, which may affect cytoskeletal organization, motility, and other important cellular functions.

During NP internalization, the ability of epithelial cells to distinguish between different NP shapes can impact NP absorption and accumulation in the cytoskeleton. For example, HeLa cervical cancer cells showed greater uptake of structured SiO_2_ NPs as cylinders compared to SiO_2_ spheres, which required the development of filopodia [51]. Ras-related C3 botulinum toxin substrate 1 (Rac1) activation and the production of F-actin stress fibers were essential for cylindrical NP uptake, whereas this activation was not present during spherical NP internalization. These findings suggest that different types of NPs can have direct toxic effects on the cell cytoskeleton, leading to changes such as the breakdown of the actin and tubulin network. Such changes can alter protein expression and disrupt normal cellular functions such as motility and adhesion. Moreover, the shape of NPs can impact their internalization and accumulation in the cytoskeleton, with cylindrical NPs being taken up more readily and requiring specific cellular processes such as filopodia development. Understanding these direct effects of NPs on the cell cytoskeleton is crucial for designing and developing safer and more effective nanomaterials for biomedical applications. This knowledge can also provide insights into potential risks and hazards associated with NP exposure.

### 2.2. Indirect Toxic Effect of NPs on Cell Cytoskeleton

Even at low concentrations of NP, changes in the cytoskeleton can precede oxidative stress and inflammation, as shown in A549 adenocarcinoma cells, which showed changes in gene expression after exposure to a low concentration of SiO_2_ (1 μg/cm^2^, 12 nm in size), which involved Rho signaling and clathrin-mediated internalization pathways [46]. Tubulin polymerization was inhibited in A549 cells after 40 h of exposure to amorphous SiO_2_ NP, indicating direct impacts on cytoskeleton components. A depolymerization process mediated by cold treatment (3 h on ice) resulted in excessive repolymerization, which was connected with a decrease in acetylated tubulin and decreased motility of cells exposed to amorphous SiO_2_ NPs [55]. Ag NPs upregulate cytokeratin 8, cytokeratin 18, and gelsolin, while actin and α- and β-tubulin are down-regulated, according to a proteomic analysis of co-cultured intestinal epithelial cells of Caco-2/TC-7 and HT29-MTX [52]. Furthermore, in the absence of cytotoxicity, an increase in interleukin-8 (IL-8) was observed, and strong dissolution of Ag (0.01%) suggested that the effects were due to NPs rather than Ag ions. SiO_2_ NPs reduced cell survival in the human keratinocyte cell line HaCaT, and those that survived exhibited morphological changes and cell cycle arrest in phase G [56]. Furthermore, these cells showed changes in chaperons gene expression, oxidative stress response and apoptosis-related proteins, and cytoskeleton-related proteins such as gelsolin-like capping protein, keratin 8 and keratin 19 [57]. ZnO NPs were ingested by endosomes and then transported to lysosomes in the same cell line [53]. After 2 h of exposure, the release of zinc ions caused cytotoxicity and actin rearrangement into cell bundles. The tubulin network produced bundles that wrapped around the nucleus and disappeared from the cell’s periphery, and, more significantly, aberrant spindles and chromosomes were dispersed throughout the cytoplasm in an uneven pattern.

Organic NPs used as drug carriers, such as dendrimers, can also cause changes in the cytoskeleton, similar to inorganic NPs. Breast cancer cells take up hyperbranched block copolymer micelles within 30 min, with internalization mediated by the clathrin and macropinocytosis pathways and NPs located around the nucleus. However, the effects on the cytoskeleton were not investigated in this study [58]. Sixth-generation cationic dendrimers have been shown to reversibly interact with actin filaments, delaying actin polymerization at low concentrations (1 μg/mL) and accelerating actin polymerization at high concentrations (≥10 μg/mL) in non-cellular systems [59]. However, further studies are needed to understand the potential toxic effects of these organic NPs on the cytoskeleton.

## 3. Effects of NPs on Cell Mechanobiology

### 3.1. Cell Mechanobiology

Mechanobiology is a field that seeks to understand how biomechanical and biophysical signals regulate cell behavior by studying the interaction between cells and their surrounding environment [60]. Cells interact with the extracellular matrix (ECM) of the tissue and respond to its changing physical properties [61,62]. ECM provides physical support, external forces, varying surface topography, and substrate stiffness [39,40,63,64], which can modify and determine cellular behaviors such as cell proliferation, differentiation, migration, invasion, and apoptosis [64,65,66,67,68]. To attach, spread, or move across a surface, cells must exert a force on the ECM, with the magnitude of the force depending on the mechanical properties of the ECM, ranging from a few to several nN [30,64]. Mechanical forces play a crucial role in directing the migration of cells [69]. The interaction between cells and the ECM is primarily maintained by components of the cell cytoskeleton, including actin structures and focal adhesion (FA) components such as PXN, myosin IIA, and focal adhesion kinase (FAK) [69].

**Figure 2:**
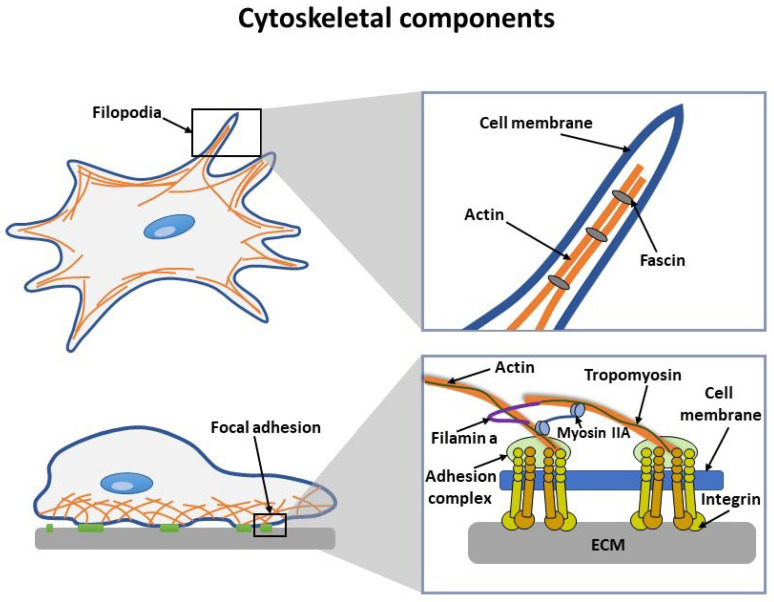
Cell cytoskeletal components for traction force production and rigidity sensing.

The cytoskeleton is a crucial component of a cell that determines its mechanical properties. It is composed of microtubules, intermediate filaments, and actin filaments. Actin filaments self-assemble to form a web-like structure that regulates intracellular forces [63,65] and provides tensile strength to the cell through parallel filaments at the leading edge, while filopodia detect environmental signals [70], as illustrated in Figure 2. The actin cytoskeleton is crucial to maintaining the mechanical properties of a cell, providing it with tensile strength and elasticity. Actin filaments self-assemble and form a web-like structure that regulates intracellular forces, while cross-linked actin structures and actin-binding proteins help maintain 3D integrity [71]. The actin structure at the leading edge of the cell forms lamellipodia and focal adhesions that attach the cell to the ECM [72]. Focal adhesions can detect changes in the environment and trigger downstream signaling pathways that regulate cell behavior [73]. During cell movement, the actin structure undergoes active remodeling, producing a traction force that enables the cell to adhere, move, and divide. Periodic changes in the actin cytoskeleton play a critical role in the formation of the cell and generating the traction force needed for cell adhesion, migration, and division [40,72,73,74].

The actin cytoskeleton, along with focal adhesion components such as myosin IIA, FAK, tropomyosin (Tmp) 2.1, and alpha-actinin, regulates the ability to sense rigidity of cells [75,76] (Figure 2). This ability is crucial when cells interact with the ECM, as it allows them to sense the mechanical properties of their environment [77]. The organized activity of focal adhesion components is necessary for cells to sense the rigidity of the ECM, with each component playing a unique role. For example, myosin IIA binds to f-actin and drives contractile structures during stiffness detection [78]. FAK, on the other hand, determines the region of the ECM that the cell can sense [79], while Tmp 2.1 regulates cell contraction and controls matrix stiffness sensing by controlling the movement steps of myosin in antiparallel actin filaments [78]. Changes in the organization of the actin scaffold or focal adhesion components can inhibit cellular activity [77]. In general, the nanoscale mechanical properties of the ECM environment can significantly influence cell behavior [80,81,82], and understanding these properties is crucial in areas such as tissue engineering and toxicity studies.

### 3.2. Methods to Investigate Cell Mechanobiology

#### 3.2.1. Cell Traction Force Measurement

**Figure 3:**
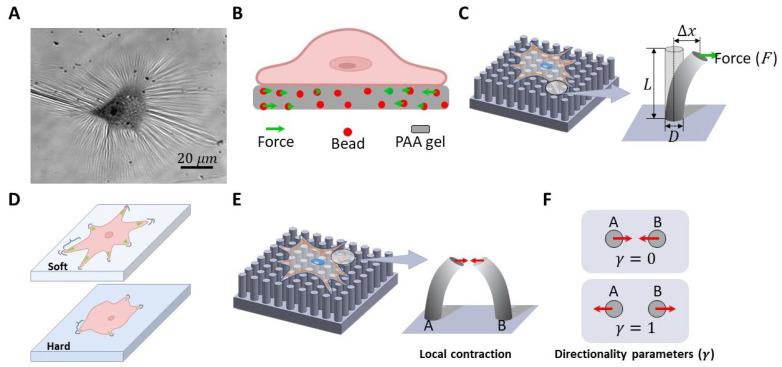
Schematic diagrams illustrating various methods for studying cell traction force and rigidity sensing. (**A**) Thin PDMS [82], (**B**) polyacrylamide (PAA) [84], and (**C**) PDMS pillars represent traction force (*F*) measurement methods [85]. ∆*x*: the displacement of the pillar from its original position. *L*: the height of the pillar. *D*: the diameter of the pillar. (**D**) Soft and hard PAA surfaces are used for rigidity sensing measurements. (**E**) PDMS submicron pillars serve as a method for measuring cellular rigidity sensing [85]. (**F**) The directionality parameter (*γ*) can be used to quantify the local contraction of a cell, where *γ* = 0 when the pillars are deflected towards each other and *γ* = 1 when the pillars are deflected in the same direction [85].

Synthetic biological techniques can aid in the study of cellular interactions with their surrounding environment, allowing for the exploration of complex signaling pathways involved in disease mechanisms [83] and nanoparticle toxicity [31,32]. The quantification of the cell traction forces has been studied using various methods. The first observation of cell traction force was made by growing cells on thin silicone rubber membranes, which caused the formation of wrinkles [82] (Figure 3A). However, due to slow processes and non-linear response of the film, this method has limitations in quantifying cell traction forces [81]. To overcome these limitations, polyacrylamide (PAA) with fluorescent beads and polydimethylsiloxane-based pillars were developed (Figure 3B). The PAA method, called traction force microscopy, uses PAA gels embedded with fluorescent beads to detect cell traction force [84]. When seeded cells deform the gel, the beads inside the gel are displaced, and the displacement of a bead from its original position is analyzed to measure cell traction force. Although the quantification of results requires careful consideration of imaging techniques, fluorescence signals, and complex mathematical models, this method offers advantages over the silicone rubber membrane method in accurately measuring cell traction forces.

Another method for measuring cell traction force is the PDMS pillar method (Figure 3C) [85]. In this method, cells are cultured on linearly arranged micrometer or sub-micrometer pillars, each of which bends when a traction force is exerted by the cell. The stiffness of the pillar (*k*) is calculated using Euler’s Bernoulli beam theory, and the displacement (∆*x*) of the pillar from its original position is calculated using the pillar tracking software [85]. In the PDMS pillar traction force measurement method, cells are cultured on identical linearly arranged micrometer or submicrometer pillars. The pillars mimic a continuous surface, providing traction force measurements that represent the entire surface. Each pillar bends when the cell exerts a traction force on it. The traction force is then quantified by multiplying the stiffness of the pillar by its displacement. The Young’s modulus (*E*) of the PDMS is about 2 MPa.

$$k=\frac{3}{64}\pi E\frac{D^{4}}{L^{3}}$$

Recent studies have shown that MNPs@SiO_2_(RITC) can alter cellular traction force production in a cell-type-dependent manner. For example, human bone-marrow-derived mesenchymal stem cells (hBM-MSCs) exhibited a significant decrease in traction force production at a concentration of 1.0 μg/μL upon MNPs@SiO_2_(RITC) treatment, while no differences were observed in the traction force production of untreated and 0.1 μg/μL treated hBM-MSC cells [25]. Similarly, the traction force of human embryonic kidney cells (HEK293) was found to increase significantly at 1 μg/μL of MNPs@SiO_2_(RITC) in another study [31]. Changes in traction force induced by MNPs@SiO_2_(RITC) were mainly due to alterations in the cell area and decreased intracellular ATP production, which could ultimately affect cell attachment and spread.

#### 3.2.2. Rigidity Sensing Measurement

One way to measure the ability of a cell to recognize the stiffness of ECM is by observing the morphological and cytoskeletal responses of cells cultured on ECM with varying stiffness [84,86]. The cell’s spreading area, aspect ratio, and length of filopodia can be quantified as morphological responses, while the cell’s actin structure and formation of focal adhesions are cytoskeletal responses. To conduct a rigidity detection study, a substrate with a stiffness ranging from 0.1 to 100 kPa can be prepared by adjusting the cross-linking ratio of the PAA gel with its curing agent, as shown in Figure 3D [84]. Similarly, by adjusting the PDMS with its curing agent ratio, a substrate can be made with a stiffness of more than 5 kPa to 2 MPa [86].

Recent studies have measured the rigidity sensing of cells by analyzing their contraction during initial spreading on submicrometer pillars. When cells sense the rigidity of the substrate, they form an adhesion complex on two or more adjacent pillars, causing them to deflect towards each other due to the actin–myosin interaction [78]. Cells deflect the adjacent pillar at an average of 60 nm during rigidity sensing, regardless of the stiffness of the pillar. However, due to the small size of the single local contraction component (<1 µm), rigidity-sensing contraction can only be detected using sub-micrometer pillars (Figure 3E). On a pillar larger than a micrometer in diameter, the rigidity sensing contraction was located on the top of a single pillar and remained undetected [85]. The directional parameter (*γ*) was calculated as the sum of the force vectors of the adjacent two pillars (*A*, *B*) divided by the sum of their magnitudes. The directionality parameter (*γ*) (Figure 3F) can be employed to quantify the local contraction of a cell, with *γ* = 0 indicating deflection of the pillars towards each other and *γ* = 1 indicating deflection of the pillars in the same direction.

$$\gamma =\frac{\sqrt{{\left(Ax+Bx\right)}^{2}+{\left(Ay+By\right)}^{2}}}{\sqrt{\left(Ax^{2}+Ay^{2}\right)}+\sqrt{\left(Bx^{2}+By^{2}\right)}}$$

**Figure 4:**
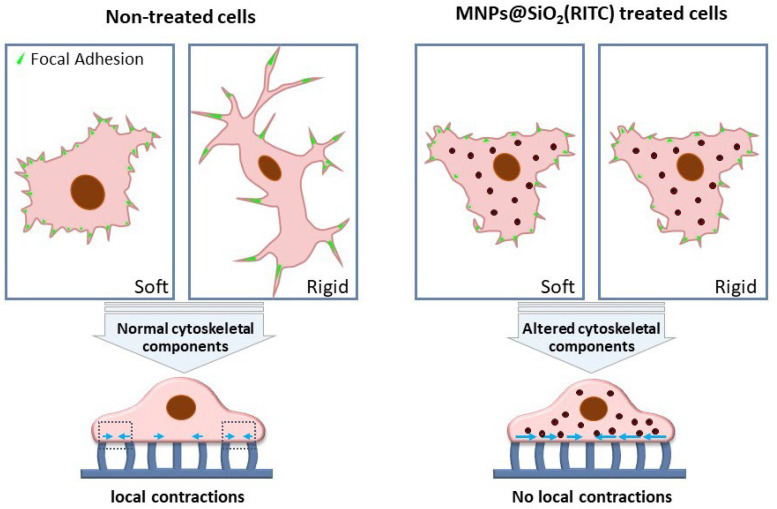
Summary of the effects of MNPs@SiO_2_(RITC) on cell rigidity sensing.

The rigidity sensing method was recently used to show the toxicity of MNPs@SiO_2_(RITC) on HEK 293 cells rigidity sensing at low concentration (0.1 µg/µL) [32]. In this study, the traditional and recent method of the rigidity detection method was applied. Using the traditional method, a soft 5 Kpa and rigid 2 Mpa flat PDMS surface was used to study the response of cells in terms of cell spreading area, aspect ratio, filopodia, and focal adhesion formation (Figure 4). Those results demonstrated that cells treated with MNPs@SiO_2_(RITC) cannot distinguish soft surfaces from rigid surfaces. The actin structure was also disrupted when MNPs@SiO_2_(RITC) enters the cell at 1 and 0.1 µg/µL on both soft and rigid surfaces. The current state of the rigidity sensing analysis method that uses submicrometer PDMS pillars showed that MNPs@SiO_2_(RITC)-treated cells cannot perform rigidity sensing contractions that occur during the initial contact of the cell substrate, indicating the loss of cell ability to sense the rigidity of the pillars. The loss of rigidity sensing is caused when MNPs@SiO_2_(RITC) damages the actin structure and cytoskeletal components, especially FSCN1 and PXN, even at 0.1 µg/µL.

This article highlights the impact of NPs on the cell cytoskeleton and mechanobiology, and the methods used to study nanotoxicity in relation to NP concentration. At lower concentrations (0.1 µg/mL), NPs generate ROS that inhibit the cell cytoskeleton, the production of traction force, and cells’ ability to sense rigidity. Changes in traction force production, rigidity sensing, and cytoskeletal components such as FSCN1 and PXN were observed after NP treatment, indicating possible cell transformation as all transformed cells display defects in their cytoskeletal components. However, no study has yet been conducted on the transformation of cells due to NPs. To further understand the effects of NPs, future research should focus on studying several types of NP based on concentration incorporated with modern microfluidic systems that mimic real organs. These studies will provide a standard for determining the toxicity of NPs.

## 4. Conclusions

The effects of NPs on cell cytoskeleton and mechanobiology have been comprehensively reviewed in this article. The toxic effects of NPs on the cytoskeletal components of cells alter the mechanobiological response of the cell, which in turn affects essential cellular functions such as cell migration, division, and proliferation. While the detailed mechanobiological studies were performed only on MNPs@SiO_2_(RITC), it is crucial to perform further studies and deep analysis since the interaction of NPs with the cell could be different due to their surface coating, size, concentrations, and chemical compositions. Incorporating rigidity sensing and traction force measurements in such studies would provide a more holistic understanding of the toxicity of NPs and aid in the development of safer NPs for various biomedical applications.

## Figures and Tables

**Table 1:** Effects of NPs on cell cytoskeletal components.

Particle Type	Toxic Effects	Conc.	Ref.
TiO_2_	Actin and tubulin breakdown, and cell adhesion	10 g/mL	[27,50]
Carbon black	Dysregulation of cell migration	10 g/mL	[50]
SiO_2_	Tubulin polymerization and decreased cell motility	200 g/mL	[46,51]
Ag NPs	Actin α- and β-tubulin downregulated	10 μg/L	[52]
ZnO	Actin rearrangement in cell bundles	10 μg/mL	[53]
Graphite nanofibers	Disrupt the actin filaments and morphological change	1 g/mL	[54]
Amorphous SiO_2_	Irregular cell shape, lamellipodia loss, distorted actin filament	-	[55]
MNPs@SiO_2_(RITC)	Decreased filopodia, lamellipodia, focal adhesion, and altered actin fibers	1 µg/µL	[25,31,32]

## Data Availability

All data generated or analyzed during this study are included in this published article.

## References

[1] Stark W.J. (2011). Nanoparticles in Biological Systems. Angew. Chem. Int. Ed..

[2] Bouallegui Y., Ben Younes R., Turki F., Oueslati R. (2017). Impact of Exposure Time, Particle Size and Uptake Pathway on Silver Nanoparticle Effects on Circulating Immune Cells in Mytilus Galloprovincialis. J. Immunotoxicol..

[3] Holzinger M., Le Goff A., Cosnier S. (2014). Nanomaterials for Biosensing Applications: A Review. Front. Chem..

[4] Mitchell M.J., Billingsley M.M., Haley R.M., Wechsler M.E., Peppas N.A., Langer R. (2021). Engineering Precision Nanoparticles for Drug Delivery. Nat. Rev. Drug Discov..

[5] Delyagina E., Schade A., Scharfenberg D., Skorska A., Lux C., Li W., Steinhoff G. (2014). Improved Transfection in Human Mesenchymal Stem Cells: Effective Intracellular Release of PDNA by Magnetic Polyplexes. Nanomedicine.

[6] Pulfer S.K., Ciccotto S.L., Gallo J.M. (1999). Distribution of Small Magnetic Particles in Brain Tumor-Bearing Rats. J. Neurooncol..

[7] Jordan A., Scholz R., Wust P., Fähling H., Felix R. (1999). Magnetic Fluid Hyperthermia (MFH): Cancer Treatment with AC Magnetic Field Induced Excitation of Biocompatible Superparamagnetic Nanoparticles. J. Magn. Magn. Mater..

[8] Yoon T.J., Kim J.S., Kim B.G., Yu K.N., Cho M.H., Lee J.K. (2005). Multifunctional Nanoparticles Possessing a “Magnetic Motor Effect” for Drug or Gene Delivery. Angew. Chem. Int. Ed..

[9] Park K.S., Tae J., Choi B., Kim Y.S., Moon C., Kim S.H., Lee H.S., Kim J., Kim J., Park J. (2010). Characterization, in Vitro Cytotoxicity Assessment, and in vivo Visualization of Multimodal, RITC-Labeled, Silica-Coated Magnetic Nanoparticles for Labeling Human Cord Blood-Derived Mesenchymal Stem Cells. Nanomedicine.

[10] Akbarzadeh A., Samiei M., Davaran S. (2012). Magnetic Nanoparticles: Preparation, Physical Properties, and Applications in Biomedicine. Nanoscale Res. Lett..

[11] Larsen B.A., Haag M.A., Serkova N.J., Shroyer K.R., Stoldt C.R. (2008). Controlled Aggregation of Superparamagnetic Iron Oxide Nanoparticles for the Development of Molecular Magnetic Resonance Imaging Probes. Nanotechnology.

[12] Patitsa M., Karathanou K., Kanaki Z., Tzioga L., Pippa N., Demetzos C., Verganelakis D.A., Cournia Z., Klinakis A. (2017). Magnetic Nanoparticles Coated with Polyarabic Acid Demonstrate Enhanced Drug Delivery and Imaging Properties for Cancer Theranostic Applications. Sci. Rep..

[13] Beck G.R., Ha S.-W., Camalier C.E., Yamaguchi M., Li Y., Lee J.-K., Weitzmann M.N. (2012). Bioactive Silica-Based Nanoparticles Stimulate Bone-Forming Osteoblasts, Suppress Bone-Resorbing Osteoclasts, and Enhance Bone Mineral Density in vivo. Nanomedicine.

[14] Dobrovolskaia M.A., McNeil S.E. (2007). Immunological Properties of Engineered Nanomaterials. Nat. Nanotechnol..

[15] Kan H., Pan D., Castranova V. (2018). Engineered Nanoparticle Exposure and Cardiovascular Effects: The Role of a Neuronal-Regulated Pathway. Inhal. Toxicol..

[16] Liu R., Liu H.H., Ji Z., Chang C.H., Xia T., Nel A.E., Cohen Y. (2015). Evaluation of Toxicity Ranking for Metal Oxide Nanoparticles via an in Vitro Dosimetry Model. ACS Nano.

[17] Wang Y., Xiong L., Tang M. (2017). Toxicity of Inhaled Particulate Matter on the Central Nervous System: Neuroinflammation, Neuropsychological Effects and Neurodegenerative Disease. J. Appl. Toxicol..

[18] Nie Z., Petukhova A., Kumacheva E. (2010). Properties and Emerging Applications of Self-Assembled Structures Made from Inorganic Nanoparticles. Nat. Nanotechnol..

[19] Shen Z., Liu T., Li Y., Lau J., Yang Z., Fan W., Zhou Z., Shi C., Ke C., Bregadze V.I. (2018). Fenton-Reaction-Acceleratable Magnetic Nanoparticles for Ferroptosis Therapy of Orthotopic Brain Tumors. ACS Nano.

[20] Sarma A., Bania R., Devi J.R., Deka S. (2021). Therapeutic Nanostructures and Nanotoxicity. J. Appl. Toxicol..

[21] Brook R.D., Franklin B., Cascio W., Hong Y., Howard G., Lipsett M., Luepker R., Mittleman M., Samet J., Smith S.C. (2004). Air Pollution and Cardiovascular Disease: A Statement for Healthcare Professionals from the Expert Panel on Population and Prevention Science of the American Heart Association. Circulation.

[22] Fu P.P., Xia Q., Hwang H.M., Ray P.C., Yu H. (2014). Mechanisms of Nanotoxicity: Generation of Reactive Oxygen Species. J. Food Drug Anal..

[23] Nel A., Xia T., Mädler L., Li N. (2006). Toxic Potential of Materials at the Nanolevel. Science.

[24] Phukan G., Shin T.H., Shim J.S., Paik M.J., Lee J.K., Choi S., Kim Y.M., Kang S.H., Kim H.S., Kang Y. (2016). Silica-Coated Magnetic Nanoparticles Impair Proteasome Activity and Increase the Formation of Cytoplasmic Inclusion Bodies in Vitro. Sci. Rep..

[25] Shin T.H., Lee D.Y., Ketebo A.A., Lee S., Manavalan B., Basith S., Ahn C., Kang S.H., Park S., Lee G. (2019). Silica-Coated Magnetic Nanoparticles Decrease Human Bone Marrow-Derived Mesenchymal Stem Cell Migratory Activity by Reducing Membrane Fluidity and Impairing Focal Adhesion. Nanomaterials.

[26] Shin T.H., Seo C., Lee D.Y., Ji M., Manavalan B., Basith S., Chakkarapani S.K., Kang S.H., Lee G., Paik M.J. (2019). Silica-Coated Magnetic Nanoparticles Induce Glucose Metabolic Dysfunction in Vitro via the Generation of Reactive Oxygen Species. Arch. Toxicol..

[27] Thai S.-F., Wallace K.A., Jones C.P., Ren H., Prasad R.Y., Ward W.O., Kohan M.J., Blackman C.F. (2015). Signaling Pathways and MicroRNA Changes in Nano-TiO2 Treated Human Lung Epithelial (BEAS-2B) Cells. J. Nanosci. Nanotechnol..

[28] Snyder R.J., Hussain S., Rice A.B., Garantziotis S. (2014). Multiwalled Carbon Nanotubes Induce Altered Morphology and Loss of Barrier Function in Human Bronchial Epithelium at Noncytotoxic Doses. Int. J. Nanomed..

[29] Pernodet N., Fang X., Sun Y., Bakhtina A., Ramakrishnan A., Sokolov J., Ulman A., Rafailovich M. (2006). Adverse Effects of Citrate/Gold Nanoparticles on Human Dermal Fibroblasts. Small.

[30] Wang H.B., Dembo M., Wang Y.L. (2000). Substrate Flexibility Regulates Growth and Apoptosis of Normal but Not Transformed Cells. Am. J. Physiol. Cell Physiol..

[31] Shin T.H., Ketebo A.A., Lee S., Kang S.H., Basith S., Manavalan B., Kwon D.H., Park S., Lee G. (2021). Decrease in Membrane Fluidity and Traction Force Induced by Silica-Coated Magnetic Nanoparticles. J. Nanobiotechnol..

[32] Ketebo A.A., Shin T.H., Jun M., Lee G., Park S. (2020). Effect of Silica-Coated Magnetic Nanoparticles on Rigidity Sensing of Human Embryonic Kidney Cells. J. Nanobiotechnol..

[33] Colin-York H., Shrestha D., Felce J.H., Waithe D., Moeendarbary E., Davis S.J., Eggeling C., Fritzsche M. (2016). Super-Resolved Traction Force Microscopy (STFM). Nano Lett..

[34] Zhang C., Wang F., Gao Z., Zhang P., Gao J., Wu X. (2020). Regulation of Hippo Signaling by Mechanical Signals and the Cytoskeleton. DNA Cell Biol..

[35] Gonzalez L., Puzzonia M.D.S., Ricci R., Aureli F., Guarguaglini G., González-Bermúdez B., Guinea G.V., Plaza G.R. (2019). Advances in Micropipette Aspiration: Applications in Cell Biomechanics, Models, and Extended Studies. Biophys. J..

[36] Bustamante C.J., Chemla Y.R., Liu S., Wang M.D. (2021). Optical Tweezers in Single-Molecule Biophysics. Nat. Rev. Methods Prim..

[37] Huang Y., Schell C., Huber T.B., Şimşek A.N., Hersch N., Merkel R., Gompper G., Sabass B. (2019). Traction Force Microscopy with Optimized Regularization and Automated Bayesian Parameter Selection for Comparing Cells. Sci. Rep..

[38] Maruthamuthu V., Sabass B., Schwarz U.S., Gardel M.L. (2011). Cell-ECM traction force modulates endogenous tension at cell-cell contacts. Proc. Natl. Acad. Sci. USA.

[39] Tan J.L., Tien J., Pirone D.M., Gray D.S., Bhadriraju K., Chen C.S. (2003). Cells Lying on a Bed of Microneedles: An Approach to Isolate Mechanical Force. Proc. Natl. Acad. Sci. USA.

[40] du Roure O., Saez A., Buguin A., Austin R.H., Chavrier P., Silberzan P., Ladoux B. (2005). Force Mapping in Epithelial Cell Migration. Proc. Natl. Acad. Sci. USA.

[41] Ghassemi S., Biais N., Maniura K., Wind S.J., Sheetz M.P., Hone J. (2008). Fabrication of Elastomer Pillar Arrays with Modulated Stiffness for Cellular Force Measurements. J. Vac. Sci. Technol. B Microelectron. Nanom. Struct. Process. Meas. Phenom..

[42] Tang X., Tofangchi A., Anand S.V., Saif T.A. (2014). A Novel Cell Traction Force Microscopy to Study Multi-Cellular System. PLoS Comput. Biol..

[43] Kamimura M., Sugawara M., Yamamoto S., Yamaguchi K., Nakanishi J. (2016). Dynamic Control of Cell Adhesion on a Stiffness-Tunable Substrate for Analyzing the Mechanobiology of Collective Cell Migration. Biomater. Sci..

[44] Limbach L.K., Li Y., Grass R.N., Brunner T.J., Hintermann M.A., Muller M., Gunther D., Stark W.J. (2005). Oxide Nanoparticle Uptake in Human Lung Fibroblasts: Effects of Particle Size, Agglomeration, and Diffusion at Low Concentrations. Environ. Sci. Technol..

[45] Shim W., Paik M.J., Nguyen D.T., Lee J.K., Lee Y., Kim J.H., Shin E.H., Kang J.S., Jung H.S., Choi S. (2012). Analysis of Changes in Gene Expression and Metabolic Profiles Induced by Silica-Coated Magnetic Nanoparticles. ACS Nano.

[46] Wang Z., Tiruppathi C., Minshall R.D., Malik A.B. (2009). Size and Dynamics of Caveolae Studied Using Nanoparticles in Living Endothelial Cells. ACS Nano.

[47] Aguilar R.C., Wendland B. (2005). Endocytosis of membrane receptors: Two pathways are better than one. Proc. Natl. Acad. Sci. USA.

[48] Meng N., Han L., Pan X., Su L., Jiang Z., Lin Z., Zhao J., Zhang S., Zhang Y., Zhao B. (2015). Nano-Mg (OH) 2-Induced Proliferation Inhibition and Dysfunction of Human Umbilical Vein Vascular Endothelial Cells through Caveolin-1-Mediated Endocytosis. Cell Biol. Toxicol..

[49] Krug H.F., Wick P. (2011). Nanotoxicology: An Interdisciplinary Challenge. Angew. Chem. Int. Ed..

[50] Vuong N.Q., Goegan P., Mohottalage S., Breznan D., Ariganello M., Williams A., Elisma F., Karthikeyan S., Vincent R., Kumarathasan P. (2016). Proteomic Changes in Human Lung Epithelial Cells (A549) in Response to Carbon Black and Titanium Dioxide Exposures. J. Proteom..

[51] Meng H., Yang S., Li Z., Xia T., Chen J., Ji Z., Zhang H., Wang X., Lin S., Huang C. (2011). Aspect Ratio Determines the Quantity of Mesoporous Silica Nanoparticle Uptake by a Small GTPase-Dependent Macropinocytosis Mechanism. ACS Nano.

[52] Georgantzopoulou A., Serchi T., Cambier S., Leclercq C.C., Renaut J., Shao J., Kruszewski M., Lentzen E., Grysan P., Eswara S. (2015). Effects of Silver Nanoparticles and Ions on a Co-Culture Model for the Gastrointestinal Epithelium. Part. Fibre Toxicol..

[53] Sudhakaran S., Athira S.S., Mohanan P. (2019). V Zinc Oxide Nanoparticle Induced Neurotoxic Potential upon Interaction with Primary Astrocytes. Neurotoxicology.

[54] Jin J., Dong Y., Wang Y., Xia L., Gu W., Bai X., Chang Y., Zhang M., Chen K., Li J. (2016). Fullerenol Nanoparticles with Structural Activity Induce Variable Intracellular Actin Filament Morphologies. J. Biomed. Nanotechnol..

[55] Gonzalez L., Puzzonia M.D.S., Ricci R., Aureli F., Guarguaglini G. (2014). Amorphous Silica Nanoparticles Alter Microtubule Dynamics and Cell Migration. Nanotoxicology.

[56] Liu W., Hu T., Zhou L., Wu D., Huang X., Ren X., Lv Y., Hong W., Huang G., Lin Z. (2017). Nrf2 Protects against Oxidative Stress Induced by SiO2 Nanoparticles. Nanomedicine.

[57] Ispanixtlahuatl-Meráz O., Schins R.P.F., Chirino Y.I. (2018). Cell Type Specific Cytoskeleton Disruption Induced by Engineered Nanoparticles. Environ. Sci. Nano.

[58] Zeng X., Zhang Y., Nyström A.M. (2012). Endocytic Uptake and Intracellular Trafficking of Bis-MPA-Based Hyperbranched Copolymer Micelles in Breast Cancer Cells. Biomacromolecules.

[59] Ruenraroengsak P., Florence A.T. (2010). Biphasic Interactions between a Cationic Dendrimer and Actin. J. Drug Target..

[60] Yadav S., Ta H.T., Nguyen N. (2021). Mechanobiology in Cardiology: Micro-and Nanotechnologies to Probe Mechanosignaling. View.

[61] Özbek S., Balasubramanian P.G., Chiquet-Ehrismann R., Tucker R.P., Adams J.C. (2010). The Evolution of Extracellular Matrix. Mol. Biol. Cell.

[62] Watt F.M., Huck W.T.S. (2013). Role of the Extracellular Matrix in Regulating Stem Cell Fate. Nat. Rev. Mol. Cell Biol..

[63] Tymchenko N., Wallentin J., Petronis S., Bjursten L.M., Kasemo B., Gold J. (2007). A Novel Cell Force Sensor for Quantification of Traction during Cell Spreading and Contact Guidance. Biophys. J..

[64] Lo C.M., Wang H.B., Dembo M., Wang Y.L. (2000). Cell Movement Is Guided by the Rigidity of the Substrate. Biophys. J..

[65] Engler A.J., Sen S., Sweeney H.L., Discher D.E. (2006). Matrix Elasticity Directs Stem Cell Lineage Specification. Cell.

[66] McBeath R., Pirone D.M., Nelson C.M., Bhadriraju K., Chen C.S. (2004). Cell Shape, Cytoskeletal Tension, and RhoA Regulate Stem Cell Lineage Commitment. Dev. Cell.

[67] Ulrich T.A., de Juan Pardo E.M., Kumar S. (2009). The Mechanical Rigidity of the Extracellular Matrix Regulates the Structure, Motility, and Proliferation of Glioma Cells. Cancer Res..

[68] Discher D.E., Janmey P., Wang Y. (2005). Tissue Cells Feel and Respond to the Stiffness of Their Substrate. Science.

[69] Carisey A., Tsang R., Greiner A.M., Nijenhuis N., Heath N., Nazgiewicz A., Kemkemer R., Derby B., Spatz J., Ballestrem C. (2013). Vinculin Regulates the Recruitment and Release of Core Focal Adhesion Proteins in a Force-Dependent Manner. Curr. Biol..

[70] Kater S.B., Davenport R.W., Guthrie P.B. (1994). Filopodia as Detectors of Environmental Cues: Signal Integration through Changes in Growth Cone Calcium Levels. Prog. Brain Res..

[71] Razinia Z., Mäkelä T., Ylänne J., Calderwood D.A. (2012). Filamins in Mechanosensing and Signaling. Annu. Rev. Biophys..

[72] Geiger B., Spatz J.P., Bershadsky A.D. (2009). Environmental Sensing through Focal Adhesions. Nat. Rev. Mol. Cell Biol..

[73] Eyckmans J., Boudou T., Yu X., Chen C.S. (2011). A Hitchhiker’s Guide to Mechanobiology. Dev. Cell.

[74] Iskratsch T., Wolfenson H., Sheetz M.P. (2014). Appreciating Force and Shape—The Rise of Mechanotransduction in Cell Biology. Nat. Rev. Mol. Cell Biol..

[75] Humphrey J.D., Dufresne E.R., Schwartz M.A. (2014). Mechanotransduction and Extracellular Matrix Homeostasis. Nat. Rev. Mol. Cell Biol..

[76] Beningo K.A., Dembo M., Kaverina I., Small J.V., Wang Y.L. (2001). Nascent Focal Adhesions Are Responsible for the Generation of Strong Propulsive Forces in Migrating Fibroblasts. J. Cell Biol..

[77] Yang B., Wolfenson H., Chung V.Y., Nakazawa N., Liu S., Hu J., Huang R.Y.-J.J., Sheetz M.P. (2019). Stopping Transformed Cancer Cell Growth by Rigidity Sensing. Nat. Mater..

[78] Wolfenson H., Meacci G., Liu S., Stachowiak M.R., Iskratsch T., Ghassemi S., Roca-Cusachs P., O’Shaughnessy B., Hone J., Sheetz M.P. (2016). Tropomyosin Controls Sarcomere-like Contractions for Rigidity Sensing and Suppressing Growth on Soft Matrices. Nat. Cell Biol..

[79] Plotnikov S.V., Pasapera A.M., Sabass B., Waterman C.M. (2012). Force Fluctuations within Focal Adhesions Mediate ECM-Rigidity Sensing to Guide Directed Cell Migration. Cell.

[80] Young J.L., Holle A.W., Spatz J.P. (2016). Nanoscale and Mechanical Properties of the Physiological Cell–ECM Microenvironment. Exp. Cell Res..

[81] Geiger B., Spatz J. (2016). Application of Synthetic Biology Approaches for Understanding Encounters between Cells and Their Microenvironment. Cell Adh. Migr..

[82] Harris A.K., Wild P., Stopak D. (1980). Silicone Rubber Substrata: A New Wrinkle in the Study of Cell Locomotion. Science.

[83] Fukuda S.P., Matsui T.S., Ichikawa T., Furukawa T., Kioka N., Fukushima S., Deguchi S. (2017). Cellular Force Assay Detects Altered Contractility Caused by a Nephritis-associated Mutation in Nonmuscle Myosin IIA. Dev. Growth Differ..

[84] Pelham R.J., Wang Y. (1997). Cell locomotion and focal adhesions are regulated by substrate flexibility. Proc. Natl. Acad. Sci. USA.

[85] Ghassemi S., Meacci G., Liu S., Gondarenko A.A., Mathur A., Roca-Cusachs P., Sheetz M.P., Hone J. (2012). Cells Test Substrate Rigidity by Local Contractions on Submicrometer Pillars. Proc. Natl. Acad. Sci. USA.

[86] Trichet L., Le Digabel J., Hawkins R.J., Vedula S.R.K., Gupta M., Ribrault C., Hersen P., Voituriez R., Ladoux B. (2012). Evidence of a Large-Scale Mechanosensing Mechanism for Cellular Adaptation to Substrate Stiffness. Proc. Natl. Acad. Sci. USA.

